# Schizophrenia genetics comes to translation

**DOI:** 10.1038/s41537-017-0011-y

**Published:** 2017-02-23

**Authors:** Enrico Domenici

**Affiliations:** Laboratory of Neurogenomic Biomarkers, Centre for Integrative Biology (CIBIO), University of Trento, Trento, Italy

The year just ended has seen substantial progress in the area of schizophrenia genomics that would deserve more than a simple editorial. Large-scale collaborative efforts initiated during the last decade^1^ are finally paying off, thanks to recent studies where researchers have mined for gold into Genome-Wide Association Study ﻿(GWAS) data. Novel insights into schizophrenia etiology justify now cautious optimism on the prospect of innovative therapeutic approaches based on genomic findings.

No more than 3 years ago, the Psychiatry Genomics Consortium (PGC) announced the identification of more than 100 risk loci for schizophrenia: their landmark paper, published in Nature in 2014,^2^ raised significant hype as well as debate within the scientific community. The genetics of schizophrenia began to appear tractable, with an amount of associated loci close to that of other well-established heritable traits,^3^ using comparable sample sizes. However, with few exceptions, historical candidate genes for schizophrenia did not make it into the list of significant loci.^4^ A huge and robust signal mapping at the MHC cluster was dominating the picture, with hundreds of genes potentially underlying the association, posing a real challenge to biological interpretation. Furthermore, as in most GWAS, all variants displayed a modest effect on disease risk as measured by odds ratios. That not withstanding, many drugs exert their therapeutic effect by acting on targets that are GWAS hits for the respective disease.^5^ The robust association identified by GWAS between common variants in the target of statins (3-Hydroxy-3-Methylglutaryl-CoA Reductase) and low-density lipoprotein levels, although modest in effect size, has been the prototypical example so far.^6^ A new finding in schizophrenia, the antipsychotic target DRD2 among the >100 PGC loci, was now strongly demanding a deeper look into the results.

At the beginning of 2016, scientists at the Broad Institute have finally unraveled the major risk locus from the PGC-schizophrenia GWAS: by an elegant integration of GWAS data with information on the effect of genetic variants on brain gene expression, they have identified a complement pathway component (C4A) as the causal gene for the robust association.^7^ Given the implication of C4A in synaptic elimination during post-natal development, this finding provides a robust genetic support to the longstanding theory of excessive synaptic pruning in schizophrenia, postulated back in 1982 (see ref. 8). Interestingly, an imbalance in synaptic pruning has been shown in other conditions, including autism^9^ and Alzheimer,^10^ warranting for the development of new therapeutics specifically targeting the complement pathway in the brain. A second locus, albeit less complex, was disentangled by integrating GWAS and brain gene expression at isoform resolution by Li *et al*.^11^ However, more than 100 loci, often involving large regions and spanning tens of genes, were still left to be deciphered.

The CommonMind Consortium (CMC) have published last November the first cornerstone of a novel integrative genomic initiative, paving the way for functional dissection of schizophrenia risk loci on a genome-wide scale.^12^ CMC aims at deciphering the genetic basis of psychiatry disorders by using brain genomic data derived from up to thousand psychiatric patients and controls post-mortem samples. Based on a genome-wide identification of brain expression quantitative trait loci and a statistical analysis of their intersection with GWAS data, the CMC members have fine-mapped the 108 loci. The results show that misregulated gene expression could, in part, explain the genetic association with schizophrenia for about 20% of the PGC loci. For five loci, a single gene was clearly identified; modeling the disease risk variants in zebrafish resulted with altered neurodevelopment for three of them. Functional analysis through eQTL-GWAS integration shows great promises: candidate causal genes and the directionality of the effect by the associated variants can be identified in parallel. Thus, the putative targets can be assessed in vitro or in model organisms for knowing in advance whether up or down-regulation would be needed for therapeutic efficacy.

The same investigation has confirmed a strong polygenic nature of schizophrenia. Much discussion is ongoing on the possibility to apply precision medicine approaches in schizophrenia.^13^ Since the above novel candidate genes were identified based on common variants, the development of “genomic” drugs targeting specific small groups of high-risk patients seems unlikely. Nevertheless, common genetic variants identified so far appears to converge on specific pathways such as immune and neuronal signaling, post-synaptic density and histone methylation.^14^ It is therefore plausible to believe that different “biological” subgroups of schizophrenia exist, with different underlying genetic etiologies mapping on specific pathways, which underlie specific dysfunctions. Indeed, GWAS are conducted on large collections assembled primarily on DSM diagnostic criteria and not on symptom endophenotypes or subtype categories. Would scientists be able to dissect the genetic nature of schizophrenia by conducting GWAS with patients stratified into subtypes, as Edwards *et al*.^15^ appear to suggest? An indirect answer might come in the very near future by the analysis of polygenic risk scores (PRS).^16^ PRS can be derived based on the sum of ‘risk alleles’ identified by reference GWAS, weighted by their effect sizes, providing an estimate of the genetic risk/burden of the disease at the individual level, for both healthy and diseased subjects. Schizophrenia PRS have been shown to associate with endophenotypes and specific symptom dimensions in schizophrenic patients^17–19^ showing how individual differences in schizophrenia overall genetic risk may result with various disease presentations. However, a more interesting picture may derive by dissecting schizophrenia cohorts by using PRS for other psychiatric disorders or related traits. For instance, PRS derived from bipolar disorder GWAS has been shown to correlate with clinical dimension of mania in schizophrenic patients, providing further evidence that clinical heterogeneity in schizophrenia can be partly ascribed to genetic factors.^20^ We predict that cross disorders PRS will be increasingly used in psychiatry genetics to attempt the dissection of diagnosis and clinical dimensions. Whether all this could become of clinical utility, or might help drug developers to stratify patient into subgroups for specific pathway drug trials, is still an open question. It looks like an aspirational but not impossible scenario. Stay tuned on schizophrenia genomics; hopefully, there’s more to come.
